# Supplementary material for: Potential Early Markers for Breast Cancer: A Proteomic Approach Comparing Saliva and Serum Samples in a Pilot Study
Source: Int J Mol Sci. 2023 Feb 19;24(4):4164. doi: 10.3390/ijms24044164 (PMC9966955; doi:10.3390/ijms24044164)
Supplement: Supplementary file 1 [file ijms-24-04164-s001.zip › supplementary figures.pdf]

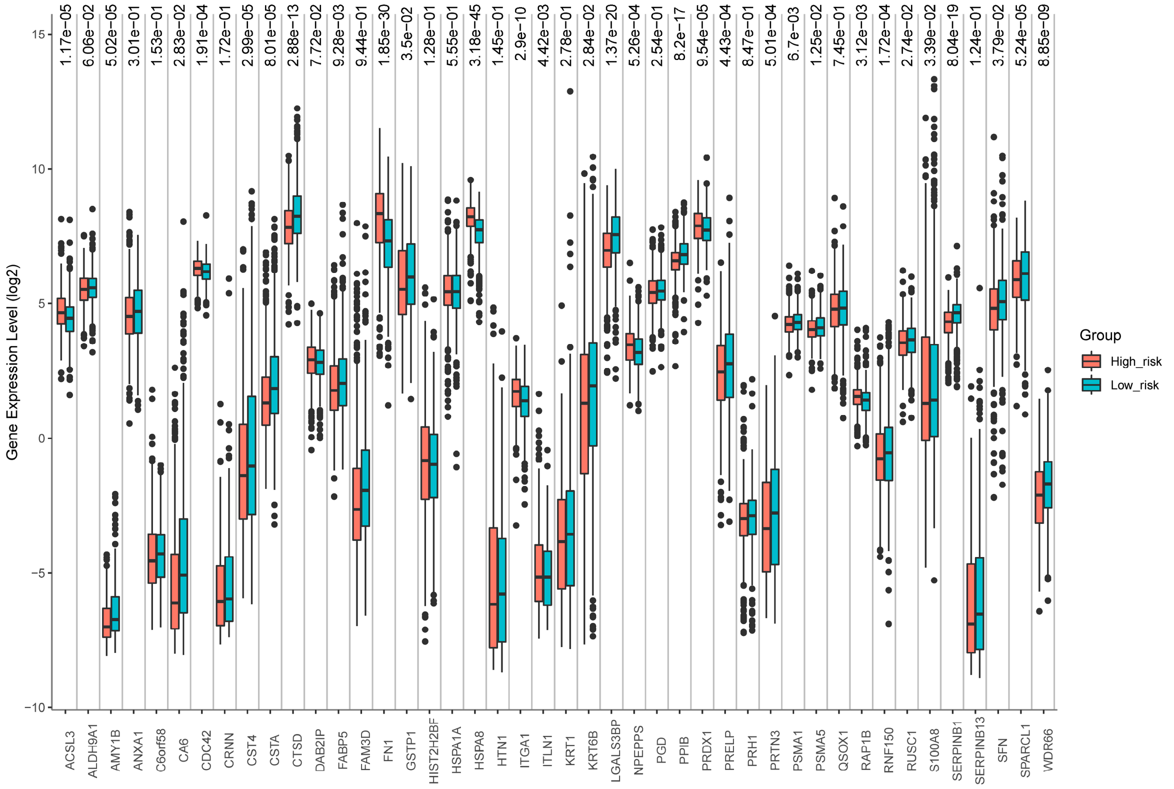

Figure S1: Prognostic performance of each gene encoding significant protein in B/N saliva group;

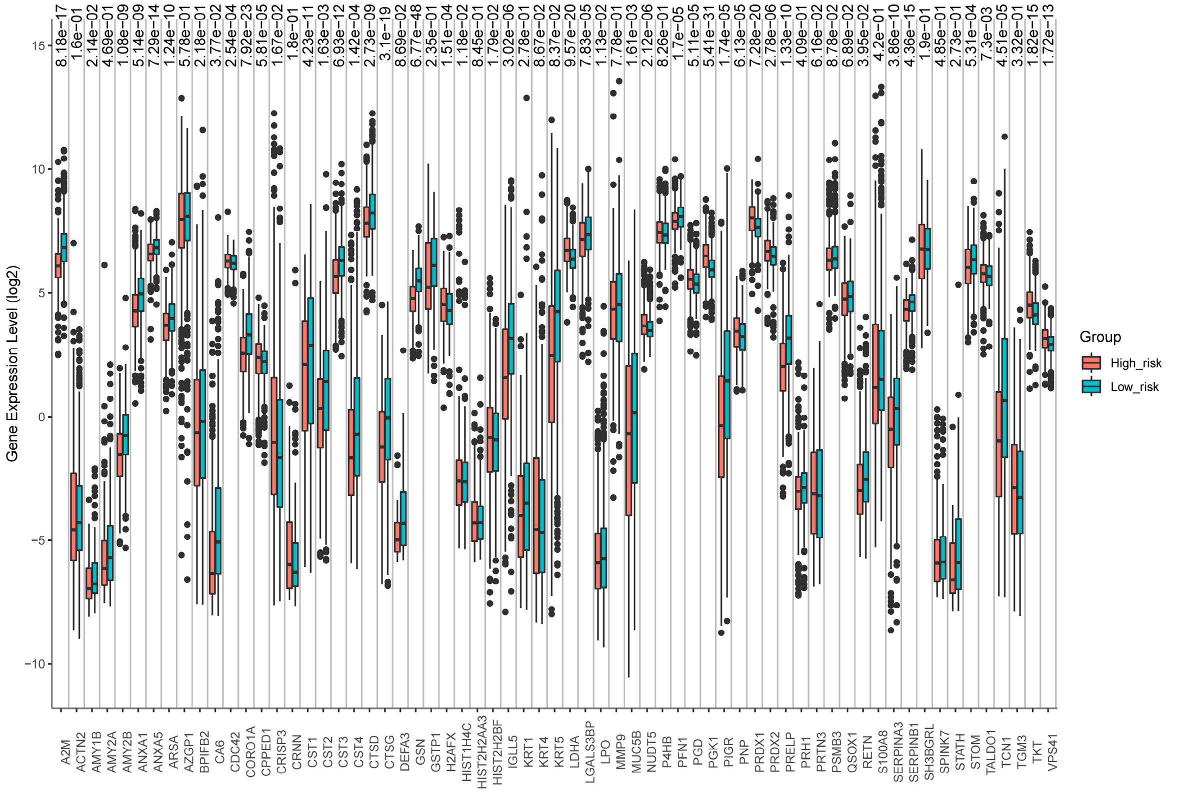

Figure S2: The prognostic performance of each gene encoding significant protein in M/N saliva group;

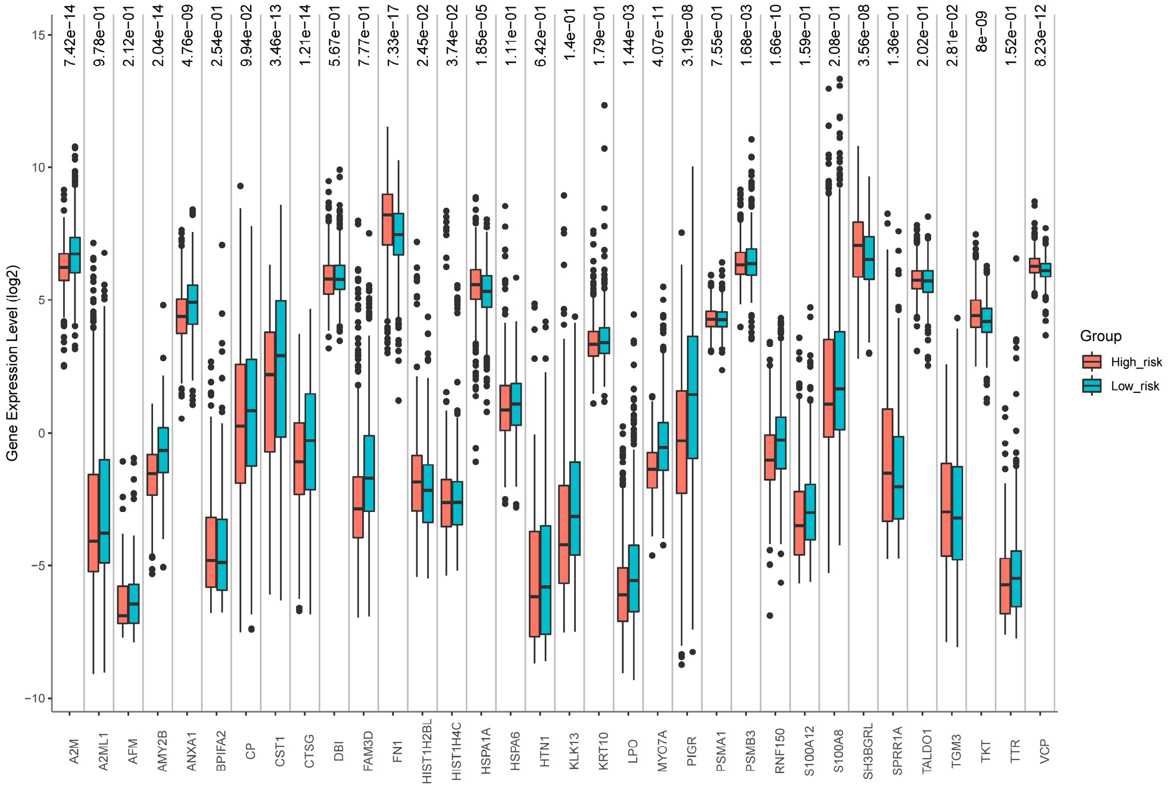

Figure S3: The prognostic performance of each gene encoding significant protein in M/B saliva group;

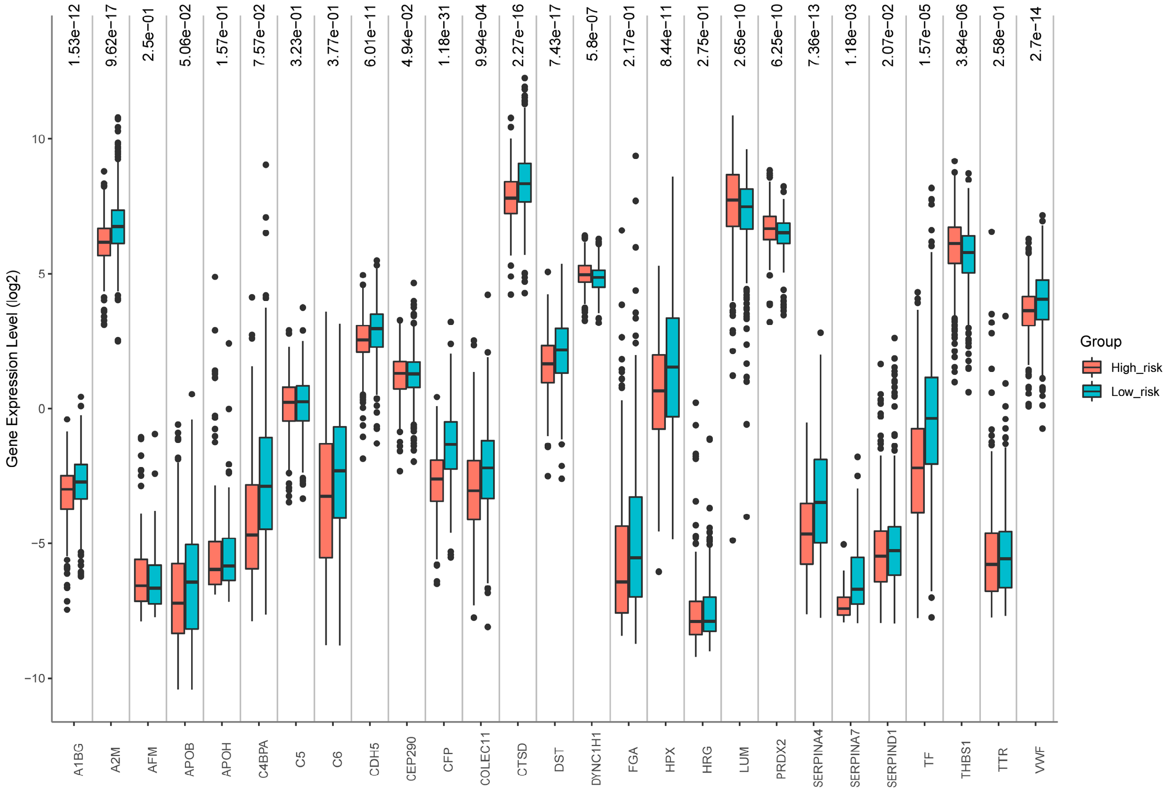

Figure S4. The prognostic performance of each gene encoding significant protein in B/N serum group;

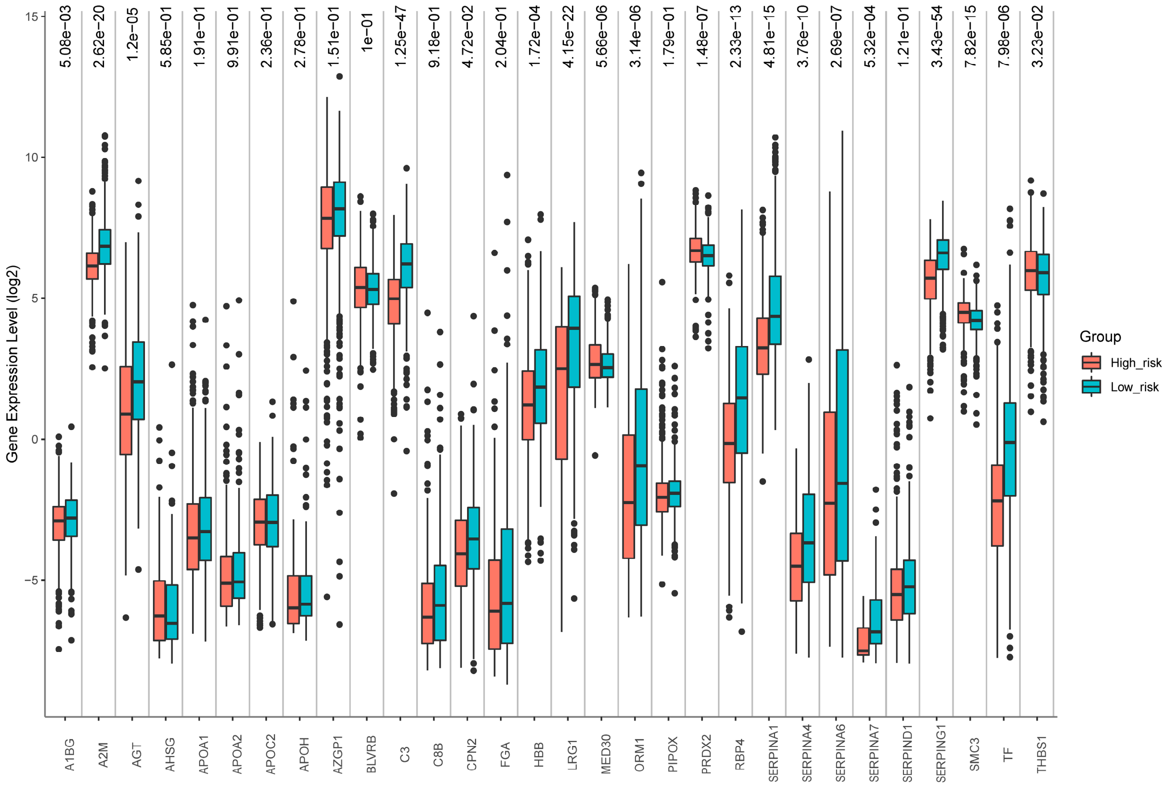

Figure S5. The prognostic performance of each gene encoding significant protein in M/N serum group;

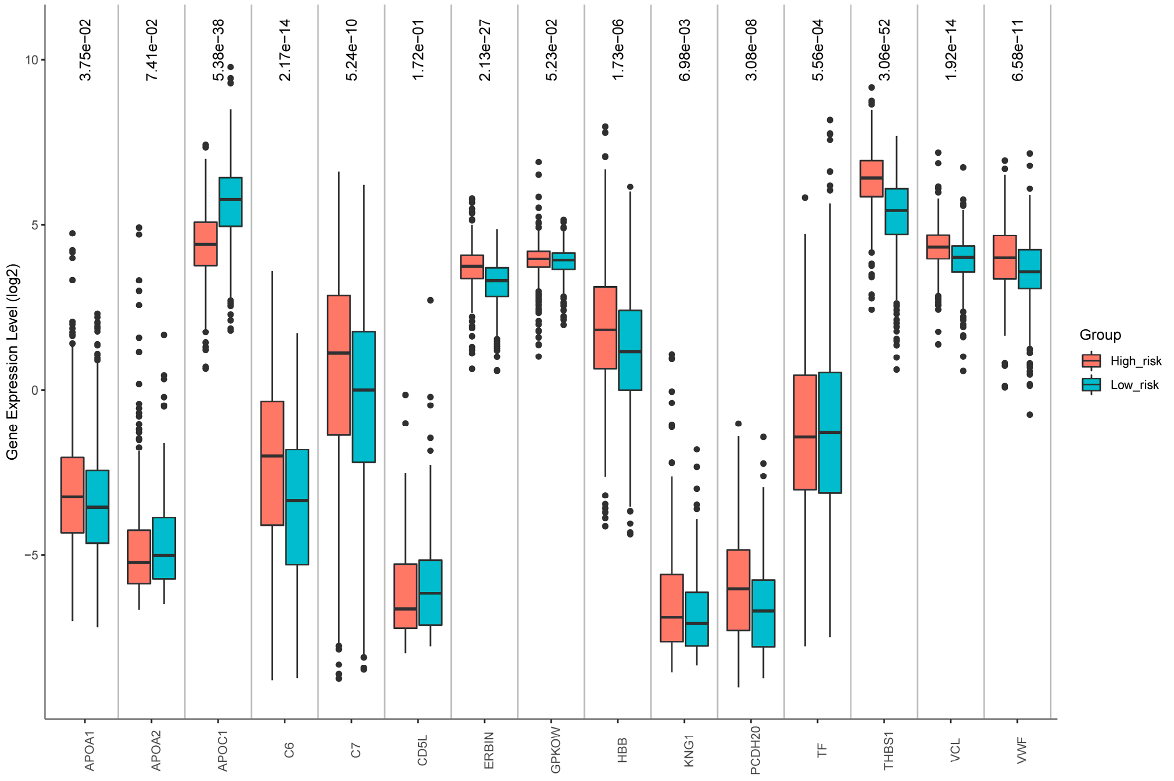

Figure S6. The prognostic performance of each gene encoding significant protein in M/B serum group;
